# Toxic leadership and employee health: exploring psychosocial stress pathways in organizational settings

**DOI:** 10.1186/s12889-026-28813-z

**Published:** 2026-08-03

**Authors:** Megren Abdullah Altassan

**Affiliations:** Department of Human Resources Management, University of Business and Technology, Jeddah, Saudi Arabia

**Keywords:** Toxic leadership, Psychosocial stress, Employee health, Organizational culture, Power distance

## Abstract

Toxic leadership poses a profound threat to employee well-being, particularly within high-context, collectivist cultures where hierarchical norms often suppress resistance. Despite growing interest globally, its psychosocial effects in Middle Eastern organizational settings remain underexplored. This qualitative study examines how toxic leadership affects the health and well-being of employees working in healthcare, business, and education organizations in Jeddah, Saudi Arabia, with particular attention to lived experiences and culturally specific coping strategies. Using an interpretivist qualitative design, semi-structured interviews were conducted in English with 42 employees from healthcare (*n* = 10), business (*n* = 12), and education (*n* = 20) organizations in Jeddah. Participants were purposively selected based on self-reported experience of or direct observation of recurrent harmful supervisory behaviors. Data were analyzed using Braun and Clarke’s six-phase thematic analysis, resulting in seven overarching themes: (1) Silent Suffering, employees experience fear, suppression, and emotional paralysis; (2) Cultural Rationalization of Abuse, submission and religious values normalize toxicity; (3) Emotional and Mental Drain, including anxiety, burnout, and psychosomatic symptoms; (4) Coping Strategies and Resistance, faith, peer support, and avoidance tactics; (5) Leadership as Untouchable Power, reinforced by favoritism and wasta; (6) Organizational Silence and HR Inaction, ineffective grievance mechanisms and distrust; and (7) Normalization and Identity Conflict, ethical erosion and emotional numbness. Findings enrich the Job Demands-Resources (JD-R) and Conservation of Resources (COR) models by contextualizing them within Saudi cultural dynamics. This study underscores the urgency for culturally aligned leadership development, mental health integration, and anti-toxic policy frameworks in the Saudi workplace.

**Supplementary Information:**

The online version contains supplementary material available at https://doi.org/10.1186/s12889-026-28813-z.

## Introduction

Toxic leadership has increasingly drawn academic attention as a destructive force within organizational settings. It encompasses a range of detrimental leadership behaviors such as abusiveness, narcissism, authoritarianism, unpredictability, and insensitivity to employee welfare [36, 39]. Unlike poor leadership due to incompetence, toxic leadership is characterized by the intentional misuse of power and manipulation to maintain dominance and control over subordinates [25]. These leaders create a hostile work environment, often eroding morale, increasing stress, and reducing overall employee engagement and productivity [27]. Globally, toxic leadership has been linked to a spectrum of negative psychosocial outcomes. These include emotional exhaustion [15], burnout [7], spillover into family relationships and family undermining [22], and reduced organizational commitment [37]. In addition, toxic leadership has been shown to impact physical health indirectly by increasing stress-related symptoms such as sleep disturbances, cardiovascular problems, and general fatigue [38]. The Gulf Cooperation Council (GCC) countries, including Saudi Arabia, possess unique socio-cultural dynamics that can shape how leadership is perceived and enacted. High power distance, hierarchical organizational cultures, and collectivist societal values make it particularly challenging for employees to confront toxic behaviors [12, 21]. In such settings, authority is rarely questioned, and loyalty often supersedes individual well-being. This dynamic often leads employees to normalize harmful leadership behaviors as a function of cultural expectations and traditional power structures [19]. In the Saudi context, factors such as “wasta” (the use of personal connections or influence), gender norms, and post-Saudization organizational transitions may further compound the experience of stress under toxic leadership [4, 9]. In light of rapid socio-economic changes under Saudi Vision 2030, leadership dynamics in public and private sectors are transforming. However, little is known about how employees experience and make sense of toxic leadership in this transitional period. Given the cultural emphasis on deference to authority and the stigma associated with discussing psychological strain, the psychosocial consequences of toxic leadership in Saudi Arabia are often underreported and poorly understood [2]. While existing studies have predominantly relied on survey-based, quantitative methodologies, such approaches often fall short in capturing the deeply embedded cultural meanings, interpersonal dynamics, and unspoken workplace norms prevalent in collectivist and high-context societies like Saudi Arabia. This study addresses that methodological gap by adopting a qualitative, interpretivist approach, enabling a richer, more culturally grounded understanding of how toxic leadership is experienced and internalized by employees.

### Problem statement

Despite the growing international literature on toxic leadership and its impact on employee well-being, empirical studies remain scarce within the Gulf and broader Middle Eastern region. Most available research has been conducted in Western or individualistic cultural contexts, where leadership structures and employee voice are fundamentally different [37]. In Saudi Arabia, especially in cities like Jeddah that serve as economic and commercial hubs, the complexity of organizational culture requires context-sensitive investigation. Moreover, post-Saudization reforms have led to significant changes in human resource practices, often intensifying pressures on both employees and managers [3]. As a result, toxic leadership may manifest more subtly through bureaucratic inefficiencies, favoritism, or inconsistent expectations. Without in-depth, qualitative insights, such phenomena may be overlooked in purely quantitative assessments. Work-related stress and coping difficulties have also been documented among professionals in Saudi Arabia, highlighting the importance of organizational approaches to employee psychological well-being [5]. However, these outcomes are rarely examined through the lens of leadership behavior. There is thus a compelling need to explore the lived experiences of employees under toxic leadership and the psychosocial stress pathways that affect their well-being in Saudi organizational contexts.

### Purpose of the study

The primary purpose of this qualitative study is to explore and interpret the lived experiences of employees working under toxic leadership within organizations in Jeddah, Saudi Arabia. By focusing on the psychosocial stress pathways, emotional exhaustion, cognitive distraction, and relational conflict, this study aims to provide a rich, contextually grounded understanding of how toxic leadership influences employees’ mental and emotional health. Additionally, the study seeks to explore how cultural and organizational factors, such as power distance, gender norms, and organizational support systems, shape, buffer, or intensify employees’ experiences of toxic leadership. By adopting an interpretivist approach and using thematic analysis of qualitative interviews, the research offers detailed insights beyond statistical associations.

### Research questions

This study is guided by the following key research questions:

1. How do employees experience and describe toxic leadership in Saudi organizations?
2. What psychosocial effects do these leadership styles have on their health and performance?
3. How do organizational and cultural factors influence or shape this experience?

These questions aim to uncover both the overt and subtle manifestations of toxic leadership as interpreted by employees themselves, especially within the unique socio-cultural fabric of Jeddah.

### Significance of the study

This study contributes to both academic and practical domains in several ways. First, it addresses a serious gap in the leadership literature by contextualizing toxic leadership within Saudi Arabia, a country undergoing significant socio-economic reform. Most leadership theories have been developed and tested in Western settings; their application in high-context, collectivist cultures remains limited [21, 23]. Second, the study provides insights into the cultural details, such as the role of “wasta,” respect for authority, and societal expectations, that influence how toxic leadership is tolerated or resisted. These insights can enrich global leadership theories, such as the Job Demands-Resources (JD-R) model [14] and Conservation of Resources (COR) theory [20], by incorporating cultural dimensions that influence psychological resource depletion and stress appraisal. Practically, the study holds implications for human resource development in Saudi organizations. As workplaces seek to modernize and align with Vision 2030, there is a pressing need for leadership development programs that foster ethical, supportive, and psychologically safe environments. This research informs HR professionals, policymakers, and organizational leaders about the importance of recognizing and addressing toxic behaviors. It also advocates for the integration of mental health initiatives and culturally grounded support systems within the organizational fabric.

### Literature review

#### Psychosocial stress and employee well-being

The psychosocial implications of toxic leadership are profound. Toxic leaders, through continuous exposure to abusive, manipulative, or indifferent behaviors, contribute to emotional exhaustion, a core dimension of burnout [26, 34]. Meta-analytic evidence indicates that destructive leadership is associated with poorer employee well-being and adverse work-related outcomes [37]. Emotional exhaustion under toxic leadership reduces one’s ability to self-regulate and engage meaningfully at work. Moreover, environments with toxic leadership are often infused with relational conflicts, further compounding workplace stress [16]. In such climates, employees may experience reduced well-being and poorer individual performance, outcomes that have been consistently associated with destructive leadership in meta-analytic research [37]. Recent studies in Middle Eastern contexts have echoed these concerns. Evidence from Saudi professional settings has documented substantial occupational stress and the use of coping strategies, underscoring the importance of contextually grounded research on employee well-being [5]. These psychosocial effects are especially significant in economies like Saudi Arabia, where workplace transformation under Vision 2030 has increased performance pressure, potentially amplifying the effect of poor leadership.

#### Understanding toxic leadership

Toxic leadership, as a construct, has gained growing attention in organizational behavior literature due to its adverse effects on both employee well-being and organizational performance. Scholars generally define toxic leadership as a pattern of destructive behaviors enacted by leaders that inflict lasting psychological harm on subordinates [25, 36]. Typologies of toxic leadership include abusive supervision, characterized by verbal hostility and humiliation [39], laissez-faire leadership, which denotes passive neglect and non-intervention [38], and tyrannical leadership, where a leader exerts excessive control to meet organizational goals, often at the cost of employee welfare [15]. Destructive leadership has been examined across diverse organizational contexts, including military institutions [33], while related nursing research has linked workplace incivility with burnout [17]. Yet, expressions of toxic leadership vary with culture. In Western societies, individualistic values may foster confrontations with toxic leaders, whereas in collectivist cultures, such behaviors may be normalized or passively accepted [21]. In high power-distance environments such as the Gulf region, hierarchical norms may make it difficult for employees to challenge authority, thereby reinforcing harmful leadership dynamics [4, 9]. Despite growing literature, there is limited qualitative insight into how these leadership styles are perceived and interpreted by employees, especially in Islamic and Arab-majority settings. Most prior studies rely on quantitative scales developed in Western contexts, such as Schmidt’s Toxic Leadership Scale, which may overlook culturally embedded meanings and experiences of harmful leadership [36].

#### Cultural context in saudi arabia

Understanding toxic leadership in Saudi Arabia requires an appreciation of sociocultural constructs such as Wasta, gender norms, collectivism, and Islamic values. Wasta, a form of social influence used to achieve goals through personal connections, can enable toxic leaders to maintain power and evade accountability, especially when they are embedded in organizational elites [13]. Comparable evidence from Pakistani higher education suggests that patronage and institutional pressure can facilitate unethical practices and weaken accountability, illustrating how informal power networks may protect misconduct within organizational settings [32]. In Saudi organizational settings, high power distance and hierarchical cultural expectations may discourage junior employees from questioning authority and reinforce top-down leadership practices [4, 9]. Gender dynamics also play a role. Female employees may be more vulnerable to psychological harm under toxic male leadership, especially in male-dominated sectors, where access to support and voice is limited [18]. These dynamics are compounded by collectivist values, which prioritize group harmony and discourage dissent, making it difficult to confront toxic leadership directly [21].

However, Islamic values, such as compassion (rahmah), justice (‘adl), and ethical leadership (amanah), offer a counterbalance. They serve as intrinsic frameworks for resilience and coping. Religious coping may serve as a culturally salient response to occupational stress in Saudi Arabia. For example, religion was among the most frequently reported coping strategies in a study of consultant physicians working in the country [5]. These values also inform organizational support systems and HR practices in culturally rooted ways. This cultural fabric makes Saudi Arabia a particularly compelling context for exploring toxic leadership qualitatively, capturing not just what happens but how it is interpreted, rationalized, or resisted by individuals within a unique social system.

#### Gaps in literature

Despite a robust global literature on toxic leadership, culturally grounded evidence from Saudi Arabia remains limited, particularly across multiple employment sectors. More broadly, leadership scholarship in the Global South remains underrepresented and contextually fragmented, underscoring the need for culturally grounded studies that account for local institutional and social conditions [31]. Although qualitative research has begun to document dysfunctional leadership experiences in Saudi healthcare organizations [1], broader cross-sector qualitative accounts remain scarce. This leads to an overgeneralization of findings and fails to capture emic perspectives, those arising from the lived experiences and cultural worldviews of participants themselves [28]. Few studies have explored how cultural factors such as tribal loyalty, religious ethics, or family expectations intersect with workplace stress under toxic leadership. Nor has much been written about how employees make sense of toxicity, whether they see it as a leadership failure, a systemic issue, or an inevitable part of work life. Without these details, HR interventions risk being superficial or culturally misaligned.

Thus, there is a key need for qualitative inquiry that listens to employee voices, examines context-bound meanings, and reveals how cultural narratives shape the experience and impact of toxic leadership. Such insights are essential for developing locally relevant leadership frameworks and for building supportive workplaces aligned with national transformation agendas like Saudi Vision 2030.

#### Conceptual framework

The conceptual framework for this study is grounded in the intersection of toxic leadership behaviors and their psychosocial effects on employees within Saudi Arabian organizational settings. Drawing from the Job Demands-Resources (JD-R) model [8], Conservation of Resources (COR) theory [20], and Social Exchange Theory [10], the framework conceptualizes how toxic leadership manifests in day-to-day interactions and how employees internalize and respond to such experiences. This study posits that toxic leadership, encompassing abusive supervision, authoritarian control, and passive neglect, acts as a primary stressor, triggering emotional and cognitive strain in employees. These stressors may manifest as emotional exhaustion, cognitive distraction, relational conflict, or other psychosomatic symptoms. Importantly, the framework incorporates organizational and cultural conditions, including *wasta*, power distance, Islamic values, organizational support, formal HR systems, peer support, and spiritual coping mechanisms. These conditions are treated as contextual influences that may shape how harmful leadership behaviors are interpreted and how employees respond to them, rather than as statistically tested moderators.

**Fig. 1:**
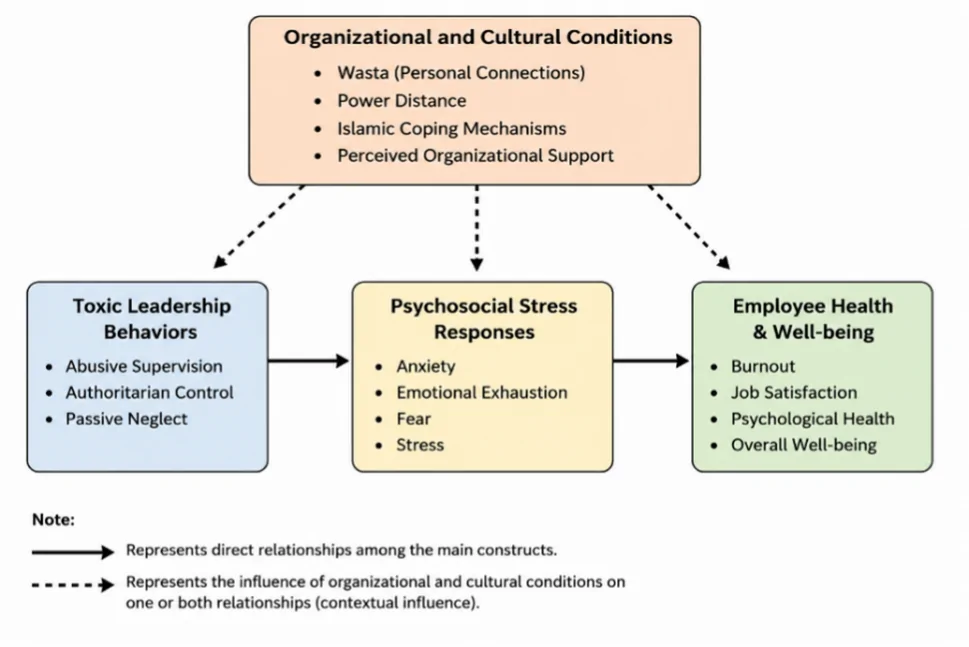
Conceptual Framework of the Study

The conceptual framework informed the interpretation of participants’ accounts but was not used as a predetermined coding template. The study employed an interpretivist qualitative exploratory design and thematic analysis to examine how employees experienced, interpreted, and coped with harmful leadership behaviors within the high-context and collectivist environment of Saudi Arabia. Figure 1 presents the relationships among leadership behaviors, psychosocial stress responses, employee health outcomes, and organizational and cultural conditions.

### Methodology

#### Research paradigm and design

This study employed a qualitative exploratory design grounded in the interpretivist paradigm. Interpretivism was considered appropriate because the study sought to understand how employees interpreted harmful leadership behaviors, psychosocial stress, workplace relationships, and coping within their particular organizational and cultural contexts. Rather than seeking to diagnose individual leaders or establish statistical relationships, the study focused on participants’ subjective experiences and the meanings they attached to those experiences.

Individual semi-structured interviews were used to generate detailed accounts of difficult supervisory interactions and their perceived consequences for employees’ psychological, emotional, physical, and occupational well-being. The reporting of the study was informed by the Consolidated Criteria for Reporting Qualitative Research (COREQ) checklist for interview-based qualitative research [40].

#### Study setting and participants

The study was conducted among employees working in healthcare, business, and education organizations in Jeddah, Saudi Arabia. Recruitment occurred at the individual employee level rather than through a formally defined sample of organizations. Consequently, the exact number of employing organizations and their public- or private-sector status were not systematically recorded. The study therefore does not make comparative claims according to organizational ownership or organization type. Organization names and other identifying details are not reported because of the sensitivity of the topic and the need to protect participant, supervisor, and institutional confidentiality.

A total of 42 employees participated in the study. Of these, 10 were employed in healthcare organizations, 12 in business organizations, and 20 in education organizations. The sample included employees from clinical, academic, administrative, technical, professional, supervisory, and managerial roles. Participants ranged in age from 25 to 55 years and included 25 men and 17 women.

The sector and gender distribution was as follows:

- Healthcare: 10 participants, comprising 7 men and 3 women;
- Business: 12 participants, comprising 7 men and 5 women; and.
- Education: 20 participants, comprising 11 men and 9 women.

#### Sampling and recruitment

Purposive criterion sampling was used to recruit employees who could provide information-rich accounts relevant to the research questions. Eligible participants were adults employed in healthcare, business, or education organizations in Jeddah who reported personal experience of, or direct observation of, recurrent harmful supervisory behavior.

For the purpose of participant eligibility, harmful supervisory behavior included repeated verbal humiliation, intimidation, authoritarian control, favoritism, threats of retaliation, deliberate exclusion, inconsistent expectations, micromanagement, or persistent managerial neglect. An isolated workplace disagreement, dissatisfaction with workload, or a single negative interaction was not considered sufficient for inclusion.

Exposure to toxic leadership was established through participants’ self-reports and their ability to describe specific recurring supervisory behaviors. No clinical or personality diagnosis was made regarding any supervisor, and no formal toxic-leadership scale was used to classify leaders. This approach was consistent with the interpretivist purpose of examining employees’ perceptions and lived experiences rather than independently determining whether a particular supervisor met a diagnostic definition of a toxic leader.

Potential participants were identified individually on the basis of the prespecified eligibility criteria and contacted directly by the researcher. Recruitment was conducted at the employee level rather than through organizational management, and the specific referral source through which each participant became known to the researcher was not systematically documented. Each potential participant received information about the study purpose, eligibility criteria, voluntary participation, confidentiality protections, and the right to decline or withdraw. Participation was not disclosed to participants’ supervisors or employers. Recruitment continued until 42 interviews had been completed, and later interviews largely repeated previously identified patterns, producing no substantively new themes relevant to the research questions.

#### Data collection

Data were collected through individual semi-structured interviews lasting approximately 25–30 min. The interviews were conducted by the sole author, an academic researcher in human resource management with knowledge of leadership and organizational behavior. The interviewer did not hold a managerial, supervisory, or performance-evaluation role over any participant.

The interviews were conducted face-to-face in Jeddah during December 2025. The interviews took place in settings that allowed participants to speak privately and confidentially. With participants’ permission, all interviews were audio-recorded and transcribed verbatim by the sole author. Before coding, each transcript was checked against its corresponding audio recording for accuracy. All names and other potentially identifying details relating to participants, supervisors, colleagues, and organizations were removed during transcription and transcript review.

The interview guide was developed from the study’s research questions and previous literature on destructive leadership, employee well-being, psychosocial stress, organizational silence, and coping. The guide began with broad questions about participants’ employment roles and relationships with supervisors before moving to specific difficult or distressing workplace interactions.

Participants were asked to describe:

1. Supervisory behaviors they found difficult or harmful;
2. The frequency and context of those behaviors;
3. Their emotional, cognitive, physical, and occupational responses;
4. The response of colleagues, senior managers, and human resource departments;
5. Cultural or organizational factors influencing their response; and.
6. Strategies used to cope with or resist the behavior.

To reduce the risk of leading participants, the interviewer did not initially label supervisors or experiences as “toxic.” Questions focused on observable behaviors and participants’ own interpretations. Neutral follow-up prompts included “Can you describe what happened?”, “Can you give an example?”, “How did that affect you?”, and “What happened after you raised or did not raise the issue?”

The interview guide was not formally piloted with members of the target population. However, its wording and sequence were reviewed before data collection to improve clarity, sensitivity, and neutrality. Evaluative wording was minimized, and participants were encouraged to provide concrete examples rather than agree with predetermined descriptions of toxic leadership. The English-language interview guide is provided as Supplementary File 1.

Participants provided informed consent before taking part. They were informed that participation was voluntary, that they could decline to answer any question, and that they could withdraw without consequence. Participant names, organization names, and identifiable details concerning supervisors or colleagues were removed. Participants were assigned identification codes from P1 to P42.

#### Language of the interviews

All interviews were conducted in English. English was selected because it was commonly used as a working language within the healthcare, business, and education settings represented in the sample, and all participants indicated that they were able to complete the interview in English. Plain and non-technical language was used throughout the interviews.

Participants were permitted to use culturally specific Arabic terms, including *wasta* and *sabr*, where these terms better represented their experiences. Such terms were retained in the analysis and explained in English to preserve their cultural meaning.

Nevertheless, conducting the interviews exclusively in English may have affected the depth and spontaneity of participants’ accounts because English was not necessarily the first or preferred language of every participant. Some culturally embedded meanings, emotional expressions, or experiences may have been more fully communicated in Arabic. This issue was considered during interpretation and is acknowledged in the study limitations.

#### Data analysis

Interview data were analyzed using the six-phase thematic analysis procedure described by Braun and Clarke [11]. The six phases included familiarization with the data, generation of initial codes, construction of candidate themes, review of themes, definition and naming of themes, and preparation of the final report.

The sole author conducted the primary coding and analysis. Coding was performed manually using a structured coding matrix. The matrix contained transcript extracts, participant codes, initial codes, working definitions, categories, candidate themes, and analytic notes. NVivo or other specialist qualitative-analysis software was not used.

The analysis began with repeated reading of the interview transcripts to develop familiarity with individual accounts and the complete dataset. Relevant sections of text were assigned initial codes representing leadership behaviors, emotional and physical responses, workplace consequences, organizational reactions, cultural interpretations, and coping strategies.

Coding was primarily inductive. The initial codes and themes were developed from participants’ accounts rather than being imposed directly from the Job Demands–Resources model, Conservation of Resources theory, or Social Exchange Theory. These theoretical frameworks were used during the later interpretive stage to explain relationships among the themes emerging from the data.

An evolving codebook was maintained throughout the analysis. The codebook included each code’s name, working definition, inclusion and exclusion boundaries, and illustrative transcript extracts. Codes expressing related meanings were grouped into broader categories. For example, codes concerning fear, suppression of complaints, retaliation, insecurity, and withdrawal were grouped during the development of the theme “Silent Suffering.”

Candidate themes were reviewed against both the coded extracts and the complete transcripts. This process assessed whether each theme was internally coherent, sufficiently distinct from other themes, supported by multiple participants, and relevant to the research questions. Overlapping themes were combined or refined, and attention was given to accounts that differed from the dominant patterns.

The analysis produced seven final themes:

1. Silent Suffering;
2. Cultural Rationalization of Abuse;
3. Emotional and Mental Drain;
4. Coping Strategies and Resistance;
5. Leadership as Untouchable Power;
6. Organizational Silence and HR Inaction; and.
7. Normalization and Identity Conflict.

A thematic map was developed to examine the relationships among the seven themes, their subthemes, and the illustrative codes.

#### Researcher reflexivity and peer debriefing

The author maintained reflexive notes during data collection and analysis. These notes documented the researcher’s assumptions, responses to participant accounts, coding decisions, changes to theme definitions, and possible influences arising from the researcher’s academic background in human resource management.

Reflexive practice was used to prevent all difficult supervisory interactions from being automatically interpreted as toxic leadership. The analysis remained focused on recurrent behaviors, participants’ interpretations, and the perceived consequences of those behaviors.

Peer debriefing was conducted with a researcher experienced in qualitative research who was not involved in participant recruitment or interviewing. The peer reviewer examined the preliminary codebook, candidate themes, thematic map, and selected supporting quotations. Discussions considered whether themes were adequately supported, whether themes overlapped, and whether alternative interpretations or contradictory cases had been considered. Decisions arising from peer debriefing were recorded in the audit trail. Final responsibility for coding and interpretation remained with the author.

#### Trustworthiness and rigor

The trustworthiness of the study was addressed using Lincoln and Guba’s [24] criteria of credibility, transferability, dependability, and confirmability.

Credibility was supported through repeated engagement with the transcripts, comparison of accounts across participants and sectors, the use of direct participant quotations, peer debriefing, and opportunities for participant clarification during the interviews. Participants were encouraged to clarify their accounts, explain culturally specific meanings, and correct any misunderstanding during and immediately after each interview. Formal post-interview member checking, including the return of transcripts or thematic summaries to participants, was not systematically conducted or documented. The study therefore does not claim formal member checking as a trustworthiness procedure.

Transferability was supported by providing detailed information about the Saudi cultural and organizational context, participant characteristics, sectors, occupational roles, and the conditions under which toxic leadership was experienced.

Dependability was supported through an audit trail documenting recruitment decisions, interview records, coding iterations, code definitions, theme development, thematic maps, reflexive notes, peer-debriefing discussions, and revisions made during the analysis. Confirmability was strengthened through reflexive documentation, peer review of the analytic structure, and the inclusion of participant quotations demonstrating the relationship between the interpretations and the underlying data.

### Ethical considerations

Ethical approval was obtained from the Deanship of Scientific Research, University of Business and Technology, Jeddah, Saudi Arabia (Reference No. 103/UBT0131/IRB.26/11/2025).

All participants received information concerning the purpose of the study, voluntary participation, confidentiality, data protection, and their right to withdraw. Informed consent was obtained before data collection. Participants were instructed not to identify their supervisors, colleagues, or organizations during the interviews. Electronic records were stored securely and were accessible only to the researcher.

### Findings

#### Participant profile

A total of 42 employees participated in the study. Of these, 10 were employed in healthcare, 12 in business, and 20 in education organizations in Jeddah. The sample included 25 men and 17 women, with ages ranging from 25 to 55 years. Participants occupied clinical, academic, administrative, technical, professional, supervisory, and managerial positions. This variation enabled the study to examine experiences of harmful leadership across sectors, employment levels, age groups, and genders. Participant characteristics are presented in Table 1.

**Table 1:** Participant Characteristics by Sector, Gender, Age Range, and Role (*N* = 42)

Participant Code	Sector	Gender	Age Range	Role
P1	Healthcare	Male	35–40	Nurse Supervisor
P2	Business	Female	30–35	HR Executive
P3	Education	Male	40–45	Lecturer
P4	Healthcare	Female	30–35	Nurse
P5	Healthcare	Male	40–45	Physician
P6	Healthcare	Male	45–50	Medical Technician
P7	Healthcare	Female	25–30	Receptionist
P8	Healthcare	Male	30–35	Clinical Officer
P9	Healthcare	Male	35–40	Pharmacist
P10	Healthcare	Female	40–45	HR Assistant
P11	Healthcare	Male	25–30	Lab Technician
P12	Healthcare	Male	50–55	Senior Doctor
P13	Business	Male	40–45	Account Manager
P14	Business	Male	30–35	Sales Executive
P15	Business	Female	25–30	Customer Service Representative
P16	Business	Male	35–40	Finance Analyst
P17	Business	Male	45–50	Operations Manager
P18	Business	Female	30–35	Marketing Officer
P19	Business	Male	25–30	IT Support
P20	Business	Female	40–45	Executive Secretary
P21	Business	Male	30–35	Logistics Officer
P22	Business	Male	50–55	General Manager
P23	Business	Female	35–40	Legal Advisor
P24	Education	Female	25–30	Teaching Assistant
P25	Education	Female	30–35	Administrative Officer
P26	Education	Male	35–40	Curriculum Developer
P27	Education	Female	40–45	School Counselor
P28	Education	Male	45–50	Principal
P29	Education	Male	30–35	Research Assistant
P30	Education	Female	35–40	Librarian
P31	Education	Male	50–55	Dean
P32	Education	Female	40–45	Registrar
P33	Education	Male	30–35	Tutor
P34	Education	Male	25–30	IT Instructor
P35	Education	Female	30–35	Student Affairs Officer
P36	Education	Male	35–40	Exam Controller
P37	Education	Male	45–50	Program Coordinator
P38	Education	Female	50–55	Academic Advisor
P39	Education	Male	40–45	Training Specialist
P40	Education	Male	25–30	Admissions Officer
P41	Education	Female	30–35	HR Officer
P42	Education	Female	25–30	Administrative Assistant

Healthcare, *n* = 10; business, *n* = 12; education, *n* = 20. Male participants, *n* = 25; female participants, *n* = 17

**Fig. 2:**
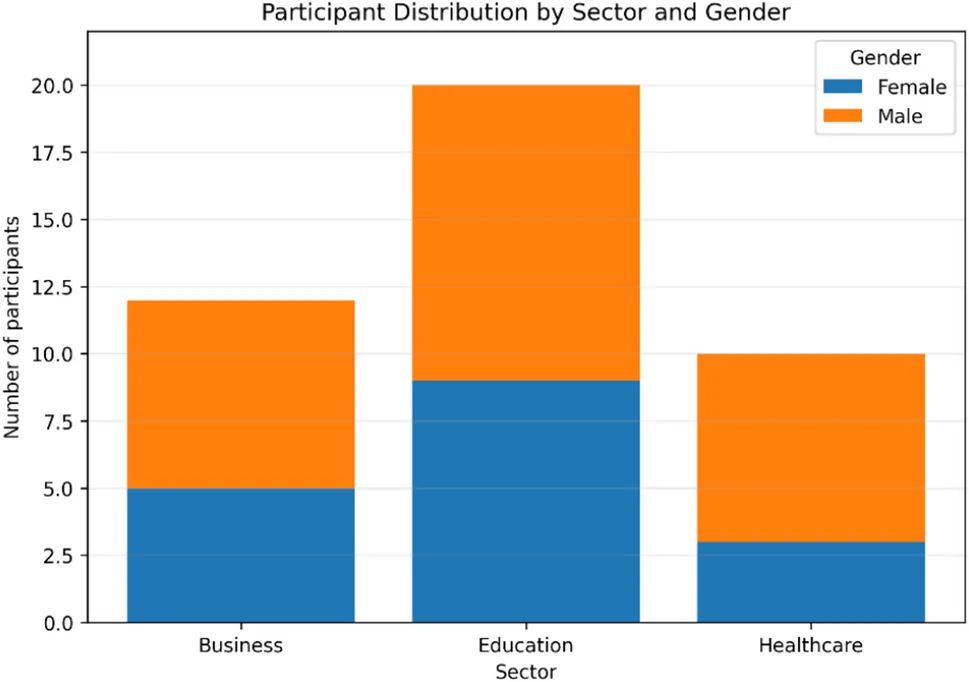
Distribution of participants by sector and gender

Figure 2 presents the distribution of participants by sector and gender. Education contributed the largest number of participants (n = 20), comprising 11 men and 9 women. Business contributed 12 participants, comprising 7 men and 5 women, while healthcare contributed 10 participants, comprising 7 men and 3 women. The sample included 25 men and 17 women.

#### Emergent themes

**Fig. 3:**
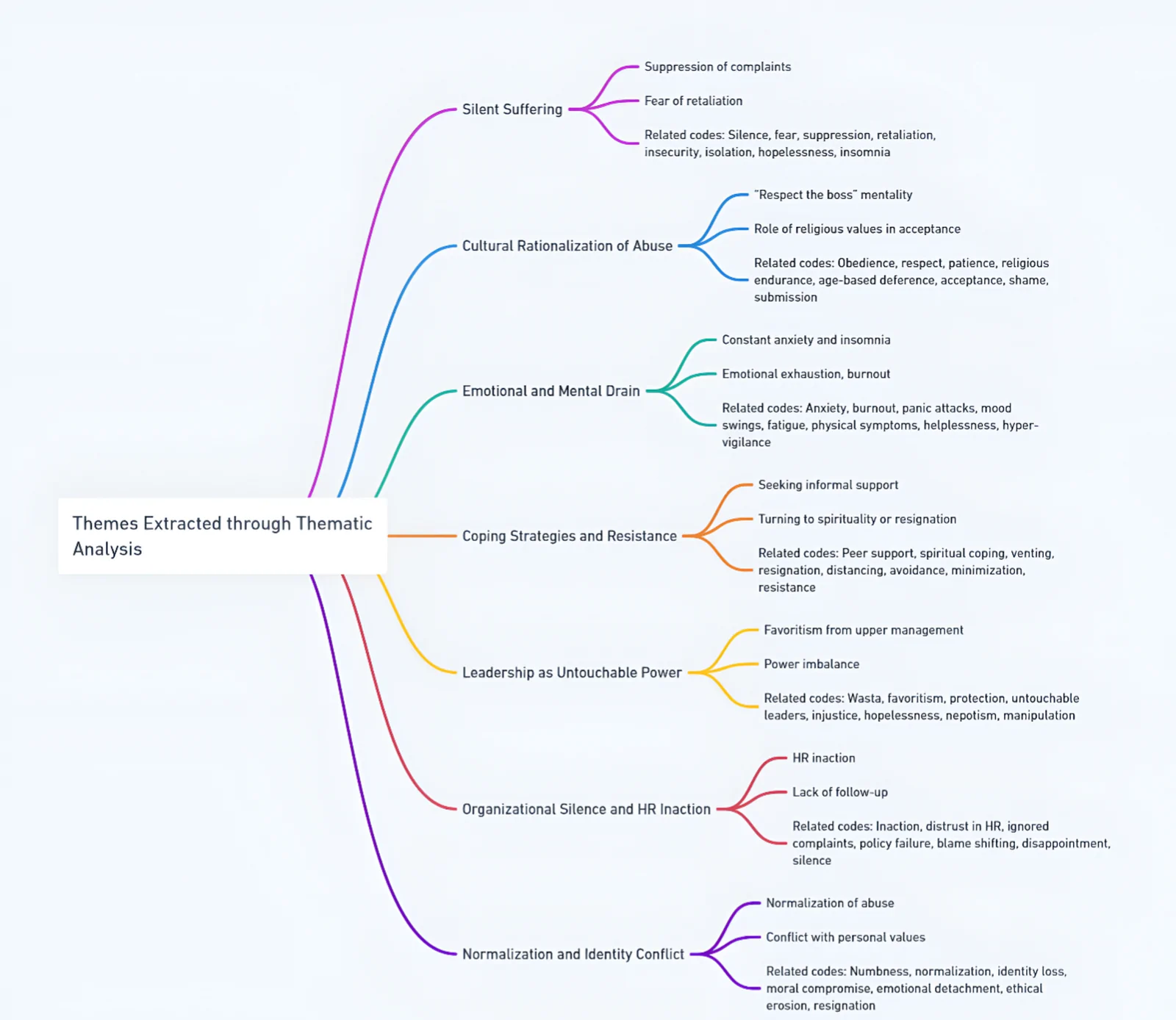
Thematic map of the seven themes

Table 2 shows that thematic analysis resulted in seven overarching themes, each with supporting subthemes. These themes provide insight into how toxic leadership is experienced, internalized, and managed by employees in the Saudi work context, as shown in the mind map in Fig. 3.

**Table 2:** Themes Extracted through Thematic Analysis

Theme	Subtheme	Supporting Codes
Silent Suffering	Suppression of complaints	Silence, suppression, isolation, hopelessness
Silent Suffering	Fear of retaliation	Fear, retaliation, insecurity, insomnia
Cultural Rationalization of Abuse	“Respect the boss” mentality	Obedience, respect, age-based deference, acceptance, shame, submission
Cultural Rationalization of Abuse	Role of religious values in acceptance	Patience, religious endurance, acceptance, submission
Emotional and Mental Drain	Constant anxiety and insomnia	Anxiety, panic attacks, mood swings, physical symptoms, hyper-vigilance, insomnia
Emotional and Mental Drain	Emotional exhaustion and burnout	Burnout, fatigue, helplessness, emotional exhaustion
Coping Strategies and Resistance	Seeking informal support	Peer support, venting, distancing, avoidance, minimization, resistance
Coping Strategies and Resistance	Turning to spirituality or resignation	Spiritual coping, religious faith, resignation
Leadership as Untouchable Power	Favoritism from upper management	Wasta, favoritism, protection, nepotism, manipulation
Leadership as Untouchable Power	Power imbalance	Untouchable leaders, injustice, hopelessness
Organizational Silence and HR Inaction	HR inaction	Inaction, ignored complaints, policy failure, blame shifting
Organizational Silence and HR Inaction	Lack of follow-up	Distrust in HR, disappointment, silence
Normalization and Identity Conflict	Normalization of abuse	Numbness, normalization, emotional detachment, resignation
Normalization and Identity Conflict	Conflict with personal values	Identity loss, moral compromise, ethical erosion

#### Theme 1: silent suffering

Participants across sectors revealed that they endured toxic leadership in silence, largely out of fear of retaliation, marginalization, or job insecurity. This silence was not due to a lack of awareness of injustice but rather an adaptive survival strategy in rigid hierarchical systems. Employees expressed internal conflict, feeling trapped between financial responsibility and psychological well-being. Silence became normalized, reinforced by colleagues, HR passivity, and cultural expectations. Over time, this led to disengagement, hopelessness, and even somatic symptoms like insomnia or chronic fatigue. The emotional burden of enduring abuse without an outlet created a toxic psychological environment, exacerbating feelings of isolation.

*“There were days I wanted to scream or walk out*,* but I just swallowed everything. My family depends on this salary*,* and I know complaining will backfire. So*,* I acted like nothing happened while I was dying inside.” (P26, Education)*

*"We all knew the manager was verbally abusive, but everyone kept quiet. Speaking up only makes things worse; you get labeled 'problematic'. You suffer silently because you have no real choice." (P13, Business) (P13, Business)*

#### Theme 2: cultural rationalization of abuse

This theme emerged from how participants justified or endured toxic behavior through deeply rooted cultural and religious beliefs. Concepts such as obedience to authority, age-based respect, and the virtue of patience were repeatedly cited. Employees often internalized abuse, believing it was a test from God or a reflection of their own shortcomings. The collectivist and hierarchical nature of Saudi society, with its emphasis on avoiding conflict and respecting seniors, further normalized toxic behaviors. These cultural filters prevented resistance and often transformed unjust treatment into spiritual endurance or personal failing.

*"We are taught since childhood, never talk back to someone older or higher. Even if the manager humiliates you, you stay respectful. It’s not right, but going against that feels like breaking the culture." (P8, Healthcare)*

*"My friends told me: 'Sabir (be patient), Allah is testing you. You’ll be rewarded.' So, I stayed. I thought maybe I deserved this. Maybe this is how growth comes. But the pain didn’t stop." (P34, Education)*

#### Theme 3: emotional and mental drain

Participants frequently described experiencing chronic stress, exhaustion, and mental breakdowns due to persistent toxic behavior. The emotional toll included panic attacks, burnout, mood swings, and withdrawal from social life. Toxic leadership not only disrupted professional function but deeply affected personal relationships and mental health. Some reported developing physical symptoms such as chest pain, migraines, or digestive issues. The recurring exposure to intimidation, micromanagement, or demeaning language created a state of hyper-vigilance and helplessness, often extending beyond the workplace. Employees began to associate the workplace with fear and mental fatigue rather than motivation.

*“I would wake up with headaches*,* heart pounding. The thought of facing him again made me sick. At work*,* I couldn’t focus; I was just counting the hours to go home.” (P19, Business)*

*“I lost interest in everything. My kids said I changed. I was anxious*,* snapping at them*,* not sleeping. I realized my job was not just stressful, it was killing my spirit.” (P6, Healthcare)*

#### Theme 4: coping strategies and resistance

Despite the psychological toll, employees demonstrated resilience through various coping strategies. Some leaned heavily on peer support or “venting circles” to process their stress. Others turned to religious faith or spiritual practices to find meaning or emotional balance. Avoidance and minimization were also common strategies, such as limiting interaction with toxic supervisors or mentally detaching during work. A few reported eventually resigning or seeking transfers. These coping methods helped reduce immediate emotional damage but often did not address the systemic issues. The line between resilience and resignation was often blurry, with coping viewed as a survival tactic rather than resistance.

*“We had this group of four*,* we’d meet for coffee after shifts just to complain and laugh it off. That space saved me. Without it*,* I would’ve broken down a long time ago.” (P41, Education)*

*“I prayed a lot. That’s what helped me breathe. Every night I asked God for strength. Eventually*,* I made peace with it and handed in my resignation. I left with my dignity intact.” (P23, Business)*

#### Theme 5: leadership as untouchable power

A strong pattern across interviews was the portrayal of toxic leaders as immune to accountability. Many were described as having personal ties with senior management or HR, commonly linked to “wasta” (favoritism or connections). This perceived protection bred hopelessness among employees who believed that no formal system would hold their superiors accountable. The imbalance of power not only enabled repeated abuse but made employees skeptical of justice. Several participants felt the organization valued the leader’s status or revenue contribution more than employee well-being. This contributed to a culture where power, not ethics, defined behavior.

*“Everyone knows he’s close to the director. He jokes with HR like they’re old friends. So*,* who will touch him? You complain*,* and they just smile and say they’ll ‘look into it.’". (P4, Healthcare)*

*“He doesn’t care because he knows nothing will happen. He’s been promoted three times despite complaints. When leadership protects toxicity*,* what hope do we have?” (P31, Education)*

#### Theme 6: organizational silence and HR inaction

Employees expressed disappointment and mistrust toward HR departments, often viewing them as extensions of management rather than neutral support systems. Complaints were often ignored, delayed, or dismissed as exaggerations. HR’s lack of visible action undermined its credibility and fueled cynicism. Some participants described HR as existing only for policy paperwork rather than employee advocacy. This systemic silence discouraged future reporting, forcing employees to either endure silently or exit the organization. The gap between HR’s stated role and perceived behavior was a recurring concern across sectors.

*“When I reported the bullying*,* HR said they’d conduct a ‘review’. Two months later*,* nothing. No feedback. No changes. I felt like I never mattered.” (P21*,* Business)*

*“They told me maybe I misunderstood him. That maybe I was being too sensitive. That crushed me. HR was my last hope*,* and they blamed me.” (P7*,* Healthcare)*

#### Theme 7: normalization and identity conflict

Several employees revealed how they gradually began to accept toxic behaviors as “just how things are” in their sector or organization. This normalization process created deep internal conflict between their values and what they were forced to tolerate. Employees wrestled with questions of self-worth, dignity, and professional identity. While some justified their endurance for financial reasons, others expressed regret and emotional fatigue. The longer they stayed, the more they questioned their ability to recognize ethical boundaries, with some fearing they were becoming numb to abuse.

*“At first*,* I was shocked when he insulted me in front of everyone. Then*,* after months*,* it became normal. That’s when I knew something was wrong with me too*,* not just him.” (P29*,* Education)*

*“I stayed for the money*,* but I lost a part of myself. I stopped reacting. I stopped caring. I became the person I promised I’d never be*,* silent in the face of wrong.” (P11*,* Healthcare)*

## Discussion

The findings of this study illuminate how toxic leadership affects employee well-being through psychosocial stress mechanisms, interpreted within the frameworks of the Job Demands-Resources (JD-R) model [8], Conservation of Resources (COR) theory [20], and Social Exchange Theory [10]. For example, the themes of *Silent Suffering* and *Emotional and Mental Drain* reflect the “emotional demands” aspect of the JD-R model, showing how prolonged toxic supervision can deplete employees’ psychological resources and lead to burnout. COR theory is evident in the constant loss of emotional energy and personal dignity reported by participants, especially in the presence of unsupportive organizational systems. The lack of reciprocity described in the *Organizational Silence and HR Inaction* theme aligns with Social Exchange Theory, where employees withdraw effort and emotional engagement when they perceive injustice and imbalance in the exchange relationship with their employer.

Culturally, these patterns are intensified in Saudi Arabia’s hierarchical and collectivist work environment. As participants noted, societal expectations of respect for authority figures and the influence of religious beliefs, such as enduring hardship as divine testing (*Cultural Rationalization of Abuse*), result in normalized suffering. Saudi cultural constructs like *wasta* (favoritism) further compound these dynamics by shielding toxic leaders from accountability, thus limiting employees’ avenues for redress. In contrast to Western settings where assertiveness is encouraged, the Saudi context often equates silence with loyalty and respect, reinforcing harmful cycles of leadership abuse and employee withdrawal.

The findings are consistent with Abalkhail’s [1] qualitative study of employees in a Saudi healthcare organization, which identified abusive behavior, marginalization, favoritism, psychological and physical harm, employee alienation, reduced commitment, and spillover into family life. The present study extends these insights across healthcare, business, and education settings and further demonstrates how cultural rationalization, organizational silence, informal power relationships, and spiritual coping shaped participants’ interpretations and responses.

### Comparison of findings with previous studies

Although toxic leadership has been extensively studied in Western settings [29, 35], relatively few qualitative studies have examined employees’ experiences in Saudi Arabia. Abalkhail [1], documented harmful leadership behaviors and their psychological, physical, occupational, and family consequences among employees in a Saudi healthcare organization. The present study supports those findings while extending the analysis to business and education settings and highlighting the roles of *sabr*, *wasta*, hierarchical expectations, organizational silence, and spiritual coping.

Although prior quantitative research has documented the adverse consequences of abusive supervision [39], participants in the present study expressed considerable skepticism toward formal organizational support mechanisms. Many participants felt that HR departments were symbolic rather than functional, lacking neutrality and reinforcing the status quo. Comparable evidence from Pakistani public universities shows that fear of retaliation, institutional silence, and distrust in inquiry committees can render formal protection policies symbolic rather than effective in practice [30]. This divergence from established literature underscores the importance of contextual, culturally embedded inquiry. While prior studies emphasize the role of organizational justice in mitigating toxic leadership [6], our findings indicate that the absence of transparent systems renders justice elusive in practice.

### Contributions to theory

This study extends existing theories by embedding them within a Saudi cultural context. It supports the JD-R and COR frameworks but details them with insights from collectivist coping. For instance, while the JD-R model typically emphasizes external job demands, our findings highlight culturally internalized demands, such as maintaining group harmony or demonstrating obedience. COR theory is expanded by showing how not only resources, but personal identity and religious faith are constantly negotiated under toxic leadership.

Furthermore, this study enriches Social Exchange Theory by demonstrating how perceived betrayal by organizational agents like HR can disrupt social contracts. In Saudi contexts, where power distance is high, the traditional “exchange” of loyalty for security is often violated, with employees expected to give more (patience, silence) without receiving support. The study also contributes to indigenous leadership theories by suggesting that imported frameworks must adapt to capture the role of spiritual coping, hierarchical fatalism, and informal power systems like *wasta* in leadership dynamics.

### Practical implications

The findings suggest three specific organizational responses. First, the themes “Organizational Silence and HR Inaction” and “Leadership as Untouchable Power” indicate the need for confidential reporting mechanisms that operate independently of employees’ immediate supervisors. Participants described complaints receiving no follow-up (P21) and leaders appearing protected through relationships with senior management or HR (P4). Organizations should therefore establish confidential hotlines, independent ombudsperson services, or third-party reporting channels. Complaints should receive written acknowledgement, protection against retaliation, a defined investigation timeline, and confidential feedback regarding the outcome.

Second, the themes “Silent Suffering,” “Cultural Rationalization of Abuse,” and “Normalization and Identity Conflict” support the introduction of mandatory leadership training. Participants explained that speaking up could result in being labelled “problematic” (P13) and that cultural expectations of respecting senior authority discouraged resistance even when humiliation occurred (P8). Leadership development should therefore distinguish legitimate authority and cultural respect from intimidation, humiliation, favoritism, and retaliation. Training should include psychological safety, emotional intelligence, ethical use of power, and culturally relevant Islamic principles such as rahmah (compassion), ‘adl (justice), and amanah (trustworthiness). These competencies should also be incorporated into managerial performance evaluations and codes of conduct.

Third, the themes “Emotional and Mental Drain” and “Coping Strategies and Resistance” demonstrate the need for accessible psychosocial support. Participants reported headaches, heart palpitations, anxiety, sleep disturbance, family strain, and emotional exhaustion (P19 and P6), while others relied mainly on informal peer support, prayer, or resignation (P41 and P23). Organizations should provide confidential bilingual counseling, employee assistance services, anonymous digital mental health support, and clear referral pathways for employees experiencing work-related psychological distress. These services should remain separate from performance evaluation systems so that employees can seek support without fear of stigma or occupational consequences.

Ultimately, organizations should periodically assess workplace culture through anonymous employee surveys, exit interviews, grievance audits, and reviews of repeated complaints concerning particular departments or supervisors. These measures would help identify patterns of normalized toxicity and evaluate whether reporting, training, and counseling interventions are producing meaningful improvements.

### Limitations

Like all qualitative studies, this research has limitations. The sample was limited to 42 participants from Jeddah, which may not fully represent all regions or sectors in Saudi Arabia. While participants were diverse in terms of gender and sector, cultural richness may vary between urban and rural areas or different cities. The education sector contributed a greater proportion of participants (*n* = 20) than the healthcare (*n* = 10) and business (*n* = 12) sectors. Consequently, the experiences and perspectives of education-sector participants may have disproportionately influenced the development and relative prominence of the themes. The findings should therefore not be interpreted as representing an equal balance of perspectives across the three sectors. Because recruitment occurred at the individual employee level, the exact number and public- or private-sector status of the employing organizations were not systematically documented. The findings should therefore be interpreted as sector-based employee accounts rather than comparisons between specific organizations or ownership categories. Formal post-interview member checking was not systematically conducted or documented; therefore, participants did not have a structured opportunity to review the final transcripts or thematic interpretations after data collection. In addition, the specific recruitment or referral source through which each participant became known to the researcher was not systematically documented, limiting a more detailed assessment of possible recruitment and self-selection bias. The interview guide was reviewed for clarity, sensitivity, and neutrality but was not formally piloted with members of the target population. Consequently, some questions may not have captured all culturally specific interpretations of difficult supervisory behavior. All interviews were conducted in English. Although English was commonly used within the professional settings represented in the study and participants were able to complete the interviews in English, it may not have been the first or preferred language of every participant. The use of English may therefore have limited the depth, spontaneity, or emotional nuance of some accounts and reduced the expression of culturally specific meanings that could have been conveyed more fully in Arabic. It may also have favored employees with greater educational or professional confidence in English. Future studies should allow participants to choose between Arabic and English and should employ bilingual interviewing and appropriate translation procedures where necessary. The primary coding and interpretation were conducted by the sole author. Although reflexive notes, peer debriefing, an audit trail, and attention to divergent accounts were used to strengthen analytic transparency, the findings remain influenced by the researcher’s interpretive position. In addition, interviews lasted approximately 25–30 min. While this duration generated focused accounts relevant to the research questions, longer or repeated interviews might have produced greater detail concerning the development and longer-term consequences of harmful supervisory relationships.

### Future research directions

Future research should explore how toxic leadership is experienced across different regions of Saudi Arabia to assess cultural variability. Longitudinal qualitative designs could better capture the cumulative psychological toll over time and evaluate whether coping mechanisms evolve. A promising direction includes mixed-methods studies that integrate survey-based instruments with narrative data for richer insights. Another avenue lies in gendered experiences of toxic leadership. While this study included male and female voices, a gender-focused inquiry may reveal distinct coping styles or power negotiations. Finally, there is scope to examine the role of religious and spiritual coping in greater depth, understanding how faith can serve both as a buffer and a barrier in confronting toxic work environments.

## Conclusion

This qualitative study demonstrates that employees who experienced or directly observed recurrent harmful supervisory behaviors perceived these experiences as deeply damaging to their psychological, emotional, and occupational well-being. Their narratives revealed recurring patterns of emotional strain, suppressed employee voice, cultural rationalization, and perceived institutional inaction. Employees across healthcare, business, and education sectors described experiences of silent suffering, where fear of retaliation, cultural norms of authority respect, and ineffective HR mechanisms left them vulnerable to ongoing psychological harm. The cultural environment, marked by hierarchical obedience, expectations of social harmony, and religious interpretations of hardship, appeared to shape and sometimes intensify participants’ reported experiences. These insights underscore an urgent need for systemic reform in organizational practices, particularly in the role of HR, to ensure safe and supportive work environments. Furthermore, mental health initiatives must be culturally sensitive and accessible, incorporating religious and societal values to be effective. Addressing toxic leadership in Saudi organizations requires more than individual resilience; it demands structural accountability, culturally aware leadership development, and the creation of safe spaces for employee voice. By integrating indigenous values with organizational reform, Saudi workplaces can cultivate psychologically safe environments that retain talent and nurture ethical leadership.

## Supplementary Information

Supplementary Material 1.

## Data Availability

Data will be provided on request from corresponding author.
