# Supplementary material for: Toxic leadership and employee health: exploring psychosocial stress pathways in organizational settings
Source: BMC Public Health. 2026 Aug 3;26:3127. doi: 10.1186/s12889-026-28813-z (PMC13721592; doi:10.1186/s12889-026-28813-z)
Supplement: Supplementary file 1 [file 12889_2026_28813_MOESM1_ESM.docx]

Supplementary File 1

Interview Guide

For the study: Toxic Leadership and Employee Health: Exploring Psychosocial Stress Pathways in Organizational Settings

| **Study setting** | Healthcare, business, and education organizations in Jeddah, Saudi Arabia |
| --- | --- |
| **Target participants** | Adult employees with personal experience of, or direct observation of, recurrent harmful supervisory behavior |
| **Interview format** | Individual, face-to-face, semi-structured interview conducted in English |
| **Approximate duration** | 25–30 minutes |

Purpose of the Guide

This guide was developed to explore how employees experience and interpret recurrent harmful supervisory behaviors, how these experiences affect their psychological, emotional, physical, and occupational well-being, and how cultural and organizational conditions shape their responses and coping strategies. Questions were used flexibly, and optional probes were applied only when clarification or greater detail was needed.

Interviewer Instructions

**•** Confirm informed consent and permission to audio-record before beginning.

**•** Remind the participant that participation is voluntary, that any question may be skipped, and that the interview may be stopped at any time.

**•** Ask the participant not to name supervisors, colleagues, or organizations. If identifying information is mentioned, redirect the participant to use a general description.

**•** Begin with broad, neutral questions. Do not initially label a supervisor, event, or relationship as “toxic.”

**•** Ask one question at a time and allow sufficient time for reflection.

**•** Use follow-up probes neutrally; do not suggest an expected answer or assume that the experience had a negative effect.

**•** Participants may use culturally specific Arabic terms, such as wasta or sabr, and should be invited to explain what those terms mean in the context of their experience.

**•** If the participant becomes distressed, offer a pause or stop the interview and remind them of their right not to continue.

Eligibility Confirmation

The following points were confirmed before the interview. These items were used only to establish study eligibility and were not treated as analytic interview questions.

| **Eligibility item** | **Confirmation** |
| --- | --- |
| Age 18 years or older | Yes / No |
| Currently or previously employed in a healthcare, business, or education organization in Jeddah | Yes / No |
| Personal experience of, or direct observation of, recurrent harmful supervisory behavior | Yes / No |
| Experience involved a recurring pattern rather than only a single disagreement or general dissatisfaction with workload | Yes / No |
| Comfortable completing the interview in English | Yes / No |
| Provides informed consent and permission for audio recording | Yes / No |

Interview Record

| **Participant code** | __________ | **Sector** | Healthcare / Business / Education |
| --- | --- | --- | --- |
| **Gender** | __________ | **Age range** | __________ |
| **Role** | __________ | **Interview date** | __________ |

Semi-Structured Interview Questions

A. Opening and Work Context

**1.** Please tell me briefly about your current role and your experience in this type of organization.

**Optional probes:** • How long have you worked in this sector or role? • What are your main responsibilities?

**2.** How would you describe your general working relationship with your current or previous supervisor?

**Optional probes:** • What was communication usually like? • How were decisions or feedback typically handled?

B. Difficult or Harmful Supervisory Experiences

**3.** Can you describe a supervisory interaction or pattern of behavior that you found particularly difficult, distressing, or harmful?

**Optional probes:** • What happened? • Who was present? • What made the experience significant for you?

**4.** Was this an isolated event, or did similar behavior occur repeatedly?

**Optional probes:** • How often did it occur? • Over what period of time? • Were there particular situations in which it was more likely to happen?

**5.** How did you interpret the supervisor’s behavior at the time?

**Optional probes:** • Did your understanding change over time? • How did you distinguish this behavior from ordinary workplace disagreement or pressure?

C. Psychosocial, Health, and Work-Related Effects

**6.** How, if at all, did these experiences affect you emotionally or psychologically?

**Optional probes:** • Did you notice anxiety, fear, anger, exhaustion, helplessness, or changes in mood? • Were there effects before or after work?

**7.** Did you notice any physical or bodily effects associated with these experiences?

**Optional probes:** • For example, changes in sleep, headaches, fatigue, appetite, chest discomfort, or digestive symptoms?

**8.** How, if at all, did these experiences affect your work?

**Optional probes:** • Concentration or decision-making? • Motivation, engagement, attendance, or performance? • Relationships with colleagues or service users?

**9.** Did the effects extend beyond the workplace?

**Optional probes:** • Family or social relationships? • Daily routines, rest, or personal well-being?

D. Organizational and Cultural Conditions

**10.** How did colleagues, senior managers, or the human resources department respond, if they became aware of the situation?

**Optional probes:** • Was support offered? • Were complaints or concerns followed up? • What happened afterward?

**11.** What influenced your decision to report, not report, confront, avoid, or tolerate the behavior?

**Optional probes:** • Concerns about retaliation or job security? • Confidence in formal procedures? • Advice from colleagues, friends, or family?

**12.** Were there any organizational or cultural expectations that shaped how you understood or responded to the situation?

**Optional probes:** • Respect for hierarchy or seniority? • Group harmony or loyalty? • Personal connections or wasta? • Gender expectations?

**13.** Did religious or spiritual beliefs play any role in how you interpreted or managed the experience?

**Optional probes:** • Prayer, patience, meaning-making, or support from others? • Did these beliefs help, complicate, or delay action?

E. Coping, Resistance, and Support

**14.** What did you do to cope with or manage the situation?

**Optional probes:** • Peer support or venting? • Avoidance or limiting contact? • Prayer or spiritual practices? • Transfer, leave, resignation, or formal reporting?

**15.** Which responses or sources of support were most helpful, and which were not helpful?

**Optional probes:** • Colleagues, family, HR, managers, counseling, or other services?

**16.** Did your coping approach change over time?

**Optional probes:** • What prompted the change? • Did you feel you were adapting, resisting, withdrawing, or becoming emotionally detached?

F. Recommendations and Closing

**17.** What could organizations do to prevent or respond more effectively to harmful supervisory behavior?

**Optional probes:** • Reporting systems? • Leadership accountability? • Training, policy enforcement, or independent review?

**18.** What kinds of support would better protect employee health and well-being in these situations?

**Optional probes:** • Confidential counseling? • Peer support? • Culturally appropriate or faith-sensitive services?

**19.** Is there anything else about your experience that you believe is important for understanding this issue?

General Neutral Probes

**•** Can you tell me more about that?

**•** Can you give a specific example?

**•** What happened next?

**•** How did you feel or respond at that point?

**•** How did you understand that situation?

**•** Was that typical or unusual?

**•** Did anyone else respond or become involved?

**•** Has your view of the experience changed over time?

Closing Script

Thank you for sharing your experiences. Before we finish, is there anything you would like to clarify, correct, or add? Please remember that your responses will be de-identified and that no names of individuals or organizations will be reported. You may contact the researcher if you later have questions about the study.

**Note.** The order and wording of questions could be adjusted to maintain a natural conversation. Not every optional probe was used in every interview. The label “toxic leadership” was not introduced at the outset; participants were first asked to describe observable behaviors and their own interpretations.
